# Radioiodinated Naphthylalanine Derivatives Targeting Pancreatic Beta Cells in Normal and Nonobese Diabetic Mice

**DOI:** 10.1155/2008/371716

**Published:** 2008-04-24

**Authors:** John K. Amartey, Yufei Shi, Ibrahim Al-Jammaz, Celestina Esguerra, Basem Al-Otaibi, Futwan Al-Mohanna

**Affiliations:** ^1^Cyclotron and Radiopharmaceuticals Department, King Faisal Specialist Hospital and Research Centre, P.O. Box 3354, Riyadh 11211, Saudi Arabia; ^2^Genetics Department, King Faisal Specialist Hospital and Research Centre, P.O. Box 3354, Riyadh 11211, Saudi Arabia; ^3^Biological and Medical Research Department, King Faisal Specialist Hospital and Research Centre, P.O. Box 3354, Riyadh 11211, Saudi Arabia

## Abstract

An imaging method capable of using a signal from pancreatic beta cells to determine their mass would be of immense value in monitoring the progression of diabetes as well as response to treatment. Somatostatin receptors (SSTRs) are expressed on beta cells and are a potential target for imaging. The main objective of this study was to investigate whether pancreatic beta cells are a target for radiolabeled naphthylalanine derivatives. The molecules were subjected to in vitro and ex vivo evaluations. Pancreatic uptake of radioactivity was lower in nonobese diabetic (NOD) mice than normal mice at all time points investigated (*P* < .05) and correlated with the number of islets in tissue sections of both control and NOD mice. Immunohistochemical and confocal fluorescent microscopic studies showed colocalization of insulin and the conjugate radioligand in the pancreas. The results demonstrated that pancreatic uptake is receptor-mediated, and that beta cells are the primary target.

## 1. INTRODUCTION

Beta cell mass (BCM) in the pancreas is a key factor in determining the amount of insulin secreted to maintain normal blood glucose level. Currently, this information can only be determined precisely at autopsy. Although BCM can be deduced from blood measurements, it has not been shown to correlate well with the dysfunction of beta cells that result from morphological and biochemical changes in the pancreas [1]. Additionally, there are no known noninvasive methods to differentiate between functional and anatomical defects in insulin secretion.

Insulin-dependent diabetes mellitus (type-1, T1D) is characterized by an autoimmune process that leads to the destruction of beta cells in individuals who are genetically predisposed to the disease [2–5]. The occult phase of the disease involves infiltration of the pancreas by mononuclear cells that begins long before the onset of the disease and progressively decreases as the BCM declines [6–12]. Consequently, after substantial loss of BCM and function, there is a need for therapeutic insulin replacement (the overt symptomatic phase of diabetes). One of the characteristics of type 2 diabetes (T2D) is insulin resistance in a setting of inadequate compensatory insulin secretory response. Additionally, several insulin production and secretion abnormalities have been described in patients with T2D [13]. Current treatment approaches for T2D include alterations in diet, commencement of an exercise program, and a variety of drugs such as insulin, biguanides, sulphonylureas (SUs), and thiazolidinediones [6]. The success of any interventional strategy may depend largely on a clear understanding of disease progression. The lack of a technique that can measure or visualize pancreatic beta cells noninvasively has left many unanswered questions regarding disease progression. Progress has been made towards imaging the endocrine pancreas, and nuclear imaging leads the way relative to other imaging modalities such as magnetic resonance imaging (MRI) and optical imaging [11]. Indium-111-oxine-labeled autologous lymphocytes [12], interleukin-2 labeled with iodine-123 and technetium-99m [14–16], technetium-99m-labeled human polyclonal immunoglobulins (Tc-99m-HIG) [17], and a specific antibody to the islet antigen have been reported as potential endocrine pancreas imaging agents [18].

Modulation of insulin secretion by antidiabetic secretagogues involves binding to high affinity sulphonylurea receptors (SURs) expressed by beta cells. Hence, SU analogs have been radiolabeled with fluorine-18 and carbon-11 and investigated as potential nuclear imaging agents. The results from these studies were not satisfactory because of negligible pancreatic uptake of the potential tracers [19–21]. Clark et al. imaged the pancreatic body with fluorine-18-benzyltrozamicol [22]. This radioligand binds specifically to neuroreceptors present on presynaptic vesicles in neurons innervating the pancreas. Simpson et al. recently reported imaging pancreatic beta cells using [^11^C]dihydrotetrabenazine ([^11^C]-DTBZ), a ligand that targets the vesicular monoamine transporter expressed on pancreatic beta cells in rodent models and in baboons [23–25]. This is a promising radiotracer and was the first of the kind to be evaluated in primates as far as we know. However, there are other potential targets that needed to be investigated targeting the beta cells.

Somatostatin receptors (SSTRs) are expressed in the endocrine pancreas, and five subtypes of human SSTRs (hSSTR1–hSSTR5) have been cloned and characterized. These receptor subtypes bind endogenous SST-14 and SST-28 with low nanomolar affinity. Reports on the selectivity of all five subtypes for synthetic SST analogs have been controversial [26–29]. However, of the known receptor subtypes, SSTR1 and SSTR5 have been colocalized to these cells [30–35]. Although there is no direct evidence of association between BCM, aging, and the status of SSTRs in diabetes, it is likely that destruction or decline in beta cell numbers would result in reduction of the densities of these receptors. We recently observed that a radioiodinated derivative of naphthylalanine, (2R)-N-(6-amino-2,2,4-tri- methylhexyl)-2-[(5-iodo(3-pyridyl))carbonylamino]-3-(2-naphthyl)propanamide (IPC-*β*-AL3) localized to the pancreas in mice [36]. In addition, we have synthesized (2R)-N-(6-amino-2,2,4-trimethylhexyl)-2-[(5-iodo(3-pyridyl))- carbonylamino]- 3 -(naphthyl)propanamide, (IPC-*α*-AL3). This paper reports further biological evaluation of the tracers as potential pancreatic beta cell detection agents.

## 2. MATERIALS AND METHODS

All chemicals and reagents were purchased from Sigma-Aldrich, Fisher, or Fluka. Radioactive samples were counted in a Packard-Canberra Cobra 5000 gamma counter. The NOD mice were purchased from Taconic, USA. Blood glucose levels were measured with a glucometer (AccuCheck, Roche). Radioligand binding data was analyzed using GraphPad Prism 4.0 (San Diego, USA).

### 2.1. Isolation of mouse pancreatic islets

The method used was adopted from a published procedure with minor modifications [37]. Briefly, the pancreata of the mice (females, aged 8 weeks) were removed immediately after death by CO_2_ asphyxiation and minced on ice in Dulbecco's Modified Eagle's Medium (DMEM). Excess fat was dissected and removed by washing with the medium. The tissue was digested with collagenase type IV (10 mg/g of wet tissue) for 45 minutes at 37°C on a shaker. The reaction was quenched by the addition of DMEM supplemented with 10% fetal bovine serum and centrifuged at 2000 rpm for 5 minutes. The cell pellet was washed three times and resuspended in the same medium containing 11 mmol/L glucose. Viability was checked by the trypan blue exclusion method, and cells were quantified using a hemocytometer. The islets were maintained in culture in the DMEM medium at 37°C and 5% CO_2_ atmosphere.

### 2.2. Stable transfection of CHO cells with somatostatin receptor subtype-1 (SSTR1)

The CHO cell line was obtained from ATCC (Rockville, MD, USA) and maintained in Ham's F12 medium supplemented with fetal calf serum (10%), 100 U/mL penicillin and 100 *μ*g/mL streptomycin in a humidified atmosphere containing 5% CO_2_. The cells were transfected with the SSTR1 construct under the control of the CMV promoter (UMR cDNA Resource Center, Rolla, MO, USA, www.cdna.org), using lipofectamine 2000 (Invitrogen, CA, USA). Forty-eight hours after transfection, the cells were cultured in the F12 medium containing 400 *μ*g/mL of G418. Three weeks after G418 selection, the stable clones were pooled for the binding experiments.

### 2.3. Radioligand binding studies

The mouse islets were isolated as described above and used immediately after recovery in DMEM medium containing glucose (3 mmol/L) for 60 minutes. The [^125^I]-Tyr^11^-SS-14 was prepared as described earlier [36]. Briefly, the islets were placed in the binding buffer system (TRIS buffer 0.15 mol/L, 0.1% BSA, 1 mmol/L CaCl_2_, 5 mmol/L MgCl_2_, 50 *μ*g/mL bacitracin, and 50 *μ*g phenylmethylsulfonylfluoride [PMSF]) in the presence of increasing concentration of the radioligand (0.02–5 nmol/L). The reactants were incubated for 1 hour, and the reaction was quenched with ice-cold buffer (1 mL). The cells were isolated using a cell harvester (Brandel, Inc. USA) and washed twice with cold-buffered saline. Cell-bound radioactivity was assayed using a gamma counter. The data were fitted to a regression function to estimate binding affinity as well as receptor density. This data was published previously [36]. The experiment was repeated using CHO cell lines separately expressing SSTR1 and SSTR2 (SSTR2 was donated by Dr. Hans-J Wester, Technical University of Munich). Displacement tests were also carried out on the two cell lines using increasing concentrations of SS-14, IPC-*α*-AL3, and IPC-*β*-AL3 (10 pmol/L–20 *μ*mol/L) and a fixed amount of [^125^I]-Tyr^11^-SS-14 (100,000 cpm).

### 2.4. Biodistribution of [^131^I]-AL3 derivatives in normal CBA/J and NOD mice

The animal experiments were carried out in accordance with institutional, national, and international guidelines for humane use of animals in research, and the local review committee approved the protocol. Each mouse (the characteristics of the mice used are shown in Table 1) was injected with 0.1 mL of the [^131^I]-IPC-*β*-AL3 solution containing 185 kBq (5 *μ*Ci) and approximately 500 ng of the labeled material. The mice were mildly anesthetized and killed by cervical dislocation at 15, 30, 60, and 120 minutes post-injection. Organs and tissues of interest were dissected, weighed, and counted in a gamma counter, calibrated for the radionuclide. The percent-injected activity per gram of tissue (per whole organ for the thyroid) was then calculated for the samples. The injected activity per mouse was estimated by counting a standard sample taken from the injectate. The experiment was repeated in 16-week-old mice. Parallel experiments were performed using NOD mice at 5, 8, and 16 weeks old. The [^131^I]-IPC-*α*-AL3 was similarly tested in 5-week-old CBA/J mice.

### 2.5. Autoradiography

In the ex vivo experiment, two control CBA/J mice and two NOD mice (17 weeks old, blood glucose >300 mg/dL) were each injected with 740 kBq (20 *μ*Ci) of [^125^I]-IPC-*β*-AL3. The animals were killed 60 minutes post injection. Pancreata were harvested and washed in 50% aqueous ethanol. The specimens were mounted on microscope slides, carefully pressed so that they were spread out evenly, and covered with a transparent adhesive tape. The samples were imaged (InstantImager, Packard) for approximately 10 minutes acquisition time. The experiment was repeated on two 26-week-old NOD mice (blood glucose >400 mg/dL), where the animals were injected with the same amount of radioactive tracer.

In a parallel experiment, ex vivo autoradiography was performed in NOD and CBA/J mice, these animals were 17 weeks old. The animals were killed, and pancreata were removed and fixed in PBS-formalin (4%, pH 7.5). The samples were processed and embedded in paraffin. Sections (5 *μ*m) were deparaffinized in xylene and rehydrated in graded ethanol (100%, 95%, 75%) to deionized water. The mounted sections were incubated with the radioiodinated IPC-*β*-AL3 (500,000 cpm) for 60 minutes at ambient temperature. The slides were washed thoroughly with binding buffer, and the samples imaged.

### 2.6. Microscopy/immunohistochemistry

To investigate the cellular location of the radiolabeled tracer observed in the biodistribution and ex vivo autoradiographic studies, immunofluorescence and histology experiments were performed. Fluorescein isothiocyanate-labeled *β*-AL3 (*β*-AL3-FITC) was prepared by incubating equimolar amounts (6 *μ*mol) of *β*-AL3 and FITC in methanol in the presence of triethylamine at ambient temperature for 60 minutes. The solid product was isolated and characterized by electrospray mass spectroscopy. Normal CBA/J and NOD mice (16 weeks old) were injected with *β*-AL3 (*β*-AL3-FITC, 1 *μ*g/mouse). One hour post administration, the animals were killed and pancreata were removed and fixed in PBS-formalin (4%, pH 7.5). The samples were embedded in paraffin, and thin sections (5 *μ*m) were cut and stained with hematoxylin and eosin. For colocalization experiments, comparable sections were deparaffinized in xylene and rehydrated in graded ethanol (100%, 95%, 75%) to deionized water, permeabilized with Triton X100 and treated with both *β*-AL3-FITC (1 *μ*g/mL) and anti-insulin antibody (mouse monoclonal IgG, Santa Cruz Biotechnology, USA) for 60 minutes at ambient temperature. They were then viewed by confocal microscopy (images were acquired using Ultra *View* LCI confocal system (PerkinElmer, USA). Images were processed using Volocity impro*vision* software (Improvision Inc. Coventry, UK) using indirect immunofluorescence of TRITC-conjugated affinity purified donkey antimouse IgG (Jackson ImmunoResearch Labs, Inc., USA). These colocalization experiments were confirmed using isolated formalin-fixed mouse islets. The islets were isolated as described earlier. To test whether the uptake of *β*-AL3-FITC by the islets and fixed sections was mediated through AL3, the experiment was repeated with fluorescein isothiocyanate alone.

### 2.7. Statistical analysis

Means were compared using student's *t*-test, and a *P*-value of < 0.05 was considered significant.

## 3. RESULTS

### 3.1. Radioligand binding studies

**Figure 1:**
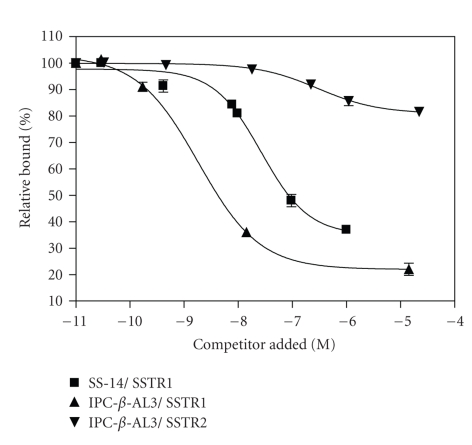
Competitive radioligand binding plots, using [^125^I]-Tyr^11^-SS-14 as the radioligand on the two cell lines demonstrated higher affinity for SSTR1 than SSTR2. (Data for IPC-a-AL3 were not included because of low specificity.)

Radioligand binding studies were performed to assess the integrity of the isolated islets. [^125^I]-Tyr-SS-14 was used in saturation as well as displacement studies. The receptor binding studies performed with SSTR1 cells revealed that a binding equilibrium was attained after 60 minutes at ambient temperature. The estimated *β*_*max*_ was 1 × 10^3^ receptors/cell. The IC_50_ values for IPC-*β*-AL3 for the SSTR1 and SSTR2 expressing cell lines were 1.83 nM and 297 nM, respectively (see Figure 1). IPC-*α*-AL3 was of low affinity and was in the *μ*M range (data not shown).

### 3.2. Biodistribution and autoradiography

**Figure 2:**
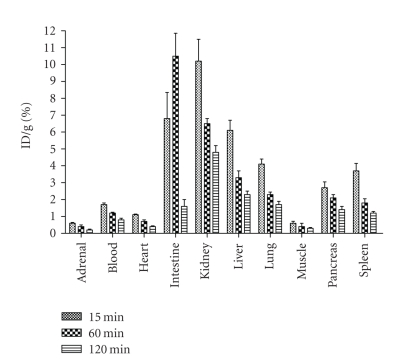
Biodistribution of [^131^I]-IPC-*β*-AL3 in NOD mice. The values are means and standard deviations of % injected radioactivity per gram. Each point represents *n* ≥ 4 mice at 5 weeks old.

The initial biodistribution was performed in sex- and age-matched nonobese diabetic (NOD) and normal CBA/J mice. A statistically significant difference in the pancreatic uptake of radioactivity was observed at 5 weeks of age. When the experiment was repeated on 8- and 16-week-old NOD and the CBA/J mice, pancreatic uptake again showed a significant difference between the two groups (*P* < 0.05). Although the uptake in the 8-week-old NOD mice was slightly higher than that of the 16-week-old NOD mice, the difference was not statistically significant (*P* < 0.05). Figure 2 shows the general biodistribution of the tracer in NOD mice at 5 weeks of age. Uptake decreased with age in the NOD mice (see Table 2), but not in the CBA/J mice.

**Figure 3:**
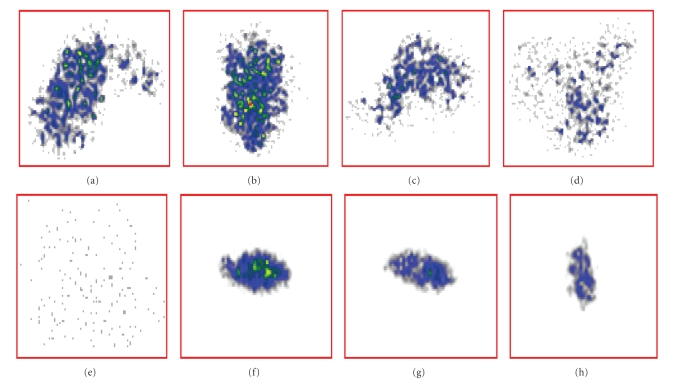
Autoradiographic images of mice pancreas injected with [^125^I]-IPC-*β*-AL3 and sacrificed 60 minutes post injection. (a) and (b) = CBA/J normal mice, (c) and (d) = 17-week-old, and (e) = 26-week-old NOD mice injected with the same quantity of radioactivity. Note the intense and scattered radioactivity in the CBA/J mice. Ex vivo autoradiograms of pancreata from normal (CBA/J) and diabetic (NOD) mice (17 weeks old). The sections were 5 *μ*m in thickness, and images were from 30 minutes of exposure. (f) and (g) = CBA/J, (h) = NOD.

**Figure 4:**
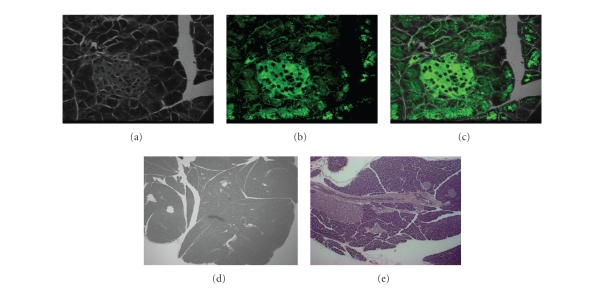
(a) Light and fluorescent micrographs of an islet, (b) the same islet labeled with AL3-FITC, (c) and the two images superimposed. (d) A hematoxylin and eosin stained section from an NOD mouse (e) and normal CBA/J pancreatic sections (e; 10× magnification). Note the presence of islets in the normal pancreas (arrows) and none in the diabetic.

Autoradiographs were obtained by imaging the pancreata of the CBA/J and NOD mice. The images demonstrated that at 16 weeks old, there was a significant difference in pancreatic uptake between the mouse strains (see Figures 3(a)–3(d)). Figure 3(e) shows the image for a 26-week-old NOD mouse. According to ex vivo autoradiograms (see Figures 3(f)–3(h)), the extent of radioactivity accumulation correlated with the number of visible islets in pancreatic sections of the CBA-J mouse under light microscope (see Figure 4(e)).

### 3.3. Microscopy/immunohistochemistry

**Figure 5:**
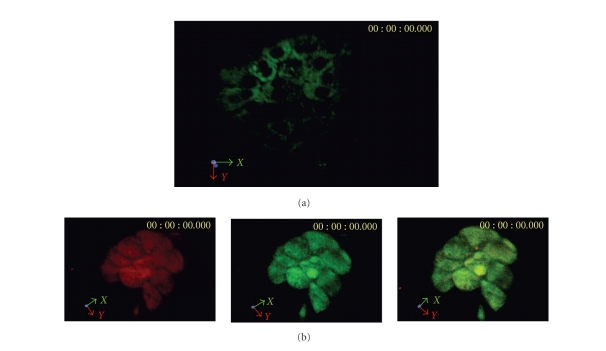
(a) A representative tomographic image of an islet stained with AL3-FITC. Note the concentration of the compound in the cell membrane. (b) Confocal laser scanning fluorescent micrographs of isolated mouse islet indirectly labeled for insulin (red) and AL3-FITC (green). The superimposed images are shown in the lower right micrograph.

To further corroborate this autoradiographic observation and the results of the radiotracer biodistribution studies, the colocalization experiment was repeated on islets isolated from normal CBA/J mice. This experiment could not be repeated in the NOD mice because enough islets could not be obtained. Additionally, hematoxylin-eosin stained and unstained sections showed no visible islets in 26-week-old NOD mice. However, there were islets of varying dimensions in the normal mice (see Figures 4(a)–4(e)). Fluorescence microscopy and histology experiments showed that the conjugate localized to the cell membrane (see Figure 5(a)). When fluorescein isothiocyanate alone was used, the uptake of dye in the pancreas was largely nonspecific. This indicated that the localization process was mediated through the AL3 portion of the conjugate. Confocal laser scanning microscopy revealed colocalization of the ligand with insulin. The insulin was localized to the cell membrane as well as in the cytoplasm, whereas the tracer was apparently cell membrane-bound (see Figure 5(b)).

## 4. DISCUSSION

Although progress has been reported in recent years [18, 22–25], such an imaging modality has not yet been fully realized. We recently reported the synthesis of a radiolabeled peptidomimetic based on (D)-2-naphthylalanine and hexanediamine and performed initial in vitro and in vivo evaluations. Our results indicated that the tracer was localized in the normal mouse pancreas [36]. We have further evaluated the radiotracers and shown that the pancreatic beta cells were the main target.

### 4.1. Radioligand binding studies

Radioligand binding studies performed on isolated mouse islets and two Chinese hamster ovary (CHO) cell lines separately expressing SSTR1 and SSTR2 yielded Kd and IC_50_ values that were consistent with previously reported values [38]. Likewise, evaluation of SSTR1 expression in SSTR1-transfected cells using a radiolabeled SST analog revealed that the SST analog affinity was in the nanomolar range, and that the number of receptors per cell was in the 10^3^ range. The results showed that the selectivity of the radioligand for SSTR1 was greater than 100 times in comparison with the SSTR2.

### 4.2. Biodistribution in mice

A statistically significant difference (*P* < .05) in the pancreatic uptake of radioactivity was observed at 5 weeks of age. This experiment was repeated when the mice were 8 and 16 weeks old, and pancreatic uptake again showed a significant difference between the CBA/J and NOD age groups. This observation may be indicative of the severity of diabetes in these mice. The biodistribution data also showed that the decrease in pancreatic uptake was not age related. We did not observe any significant difference in the control mice between 5 to 16 weeks of age. However, as a potential imaging agent for the beta cells the relatively higher uptake in the liver, intestine, and the spleen is a major drawback because of the proximity of these organs to the pancreas. This potential interference may be minimized by making the tracer less lipophilic. One approach will be to prepare a metal (Tc-99m) chelate by attaching a chelator to the nonessential part of the molecule.

### 4.3. Autoradiography

Radioactivity was localized and concentrated in the pancreas in a pattern similar to the anatomical structure of islets in the pancreas. Our observations reflected the typical scattered appearance of islets in the pancreas surrounded by nonendocrine tissue [18]. Although we did not quantify the beta cells in the islets in the fixed pancreatic sections, visual counting under a light microscope showed that there were several islets of varying sizes. This observation correlated with the extent of radioactivity accumulation in the ex vivo autoradiograms.

### 4.4. Histology and microscopic studies

The heterogeneous distribution pattern of radioactivity within the pancreas seemed to follow the variation of islet sizes and densities seen in the hematoxylin-eosin stained sections. However, the NOD mice showed pancreatic uptake slightly above background radioactivity. This observation may be due to nonspecific binding and confirmed what we observed in the previous blocking studies [36]. The uptake was increased 2-3 folds in the normal compared with the NOD mice at 26 weeks of age. The lack of substantial radioactivity in the pancreata of NOD mice was confirmed by the paucity of visible islets in the pancreatic sections of these mice. Nonetheless, the apparent high uptake in the CBA/J mice cannot be explained by perfusion alone since the circulating radioactivity in the blood was low at later time points in the biodistribution experiments. Additionally, if the uptake in the pancreas was due to inflammation during immune cell infiltration of the pancreas (associated with leaky vasculature), then the NOD mice would be expected to show a higher uptake than normal CBA/J mice, but this was not observed.

Fluorescence microscopy and histology experiments indicated that the localization process was mediated through the AL3 portion of the conjugate. The insulin was localized to the cell membrane as well as in the cytoplasm; whereas the tracer was apparently cell membrane-bound. There are two possible explanations for this result. It may be due to the presence of insulin bound to its receptors on the plasma membranes of the islets, or due to leakage of insulin, which was then fixed to the plasma membrane during tissue processing, from the secretory granules. Nonetheless, these data suggest that the tracer localizes to the islets, and the beta cells are its target. Consistent with our prior result, when the colocalization experiment was repeated on isolated mouse islets, images of live cells demonstrated that insulin was present in the cell membrane as well as in the cytoplasm, while the tracer was bound to the cell membrane. These radioligand binding and live cell imaging data indicate that the uptake of radioactivity in the pancreas was receptor-mediated and that SSTR1, but not SSTR2, was largely targeted.

Collectively, the data show that the tracer binds to beta cells. The extent of localization may be indicative of the stage of diabetes development in these mice. Although the results showed a reasonable contrast between normal and diabetic pancreata, in our estimation, it was not high enough for good in vivo imaging. The data also suggest SSTR1 involvement in the pancreatic uptake of the tracer; however, there appeared to be nonspecific binding as well. To overcome this shortcoming, this molecule may need to be optimized to obtain a higher signal-to-background ratio by decreasing the lipophilicity. This investigation is already in progress in our laboratory.

## 5. CONCLUSION

Our results indicate that this novel radiotracer targets beta cells in the mouse pancreas. We can also infer that decreased pancreatic uptake of the radiotracer in NOD mice may be directly related to the progressive destruction of beta cells in these mice.

## Figures and Tables

**Table 1:** Characteristics of mice used in the biodistribution studies. Values are means ± standard deviations for *n* ≥ 4. ND = not determined.

Age (weeks)	CBA/J		NOD	
	Weight (g)	Glucose (mg/dL)	Weight (g)	Glucose (mg/dL)
5	22 ± 3	70–100	22 ± 3	88–160
8	ND	ND	25 ± 2	120–180
16	27 ± 3	70–90	26 ± 2	260–450

**Table 2:** Comparative biodistribution of [^131^I]-IPC-*β*-AL3 in normal CBA/J and NOD mice with age, 60 minutes post injection. Values are means ± standard deviations of % ID/g, where *n* = ≥ 4 per time point. The small intestine was measured with its contents. The value for the thyroid is for the whole organ (% ID/organ).

	Mouse groups by age (weeks) and strain					
	5		8		16	
Organ/Tissue	CBA/J	NOD	CBA/J	NOD	CBA/J	NOD
Blood	0.7 ± 0.1	1.2 ± 0.1	0.9 ± 0.2	0.6 ± 0.1	0.5 ± 0.1	0.3 ± 0.1
Heart	1.8 ± 0.6	0.7 ± 0.2	2.3 ± 0.7	1.0 ± 0.4	1.2 ± 0.2	0.5 ± 0.1
Intestine (small)	20.7 ± 13.3	10.5 ± 2.7	7.4 ± 1.9*	3.3 ± 1.5*	12.5 ± 3.9	17.1 ± 5.1
Kidney	24.6 ± 7.9	6.5 ± 0.6	13.2 ± 3.3	6.5 ± 1.6	14.4 ± 4.3	5.3 ± 1.1
Liver	3.9 ± 0.6	3.3 ± 0.8	4.4 ± 0.4	3.6 ± 1.4	2.7 ± 0.6	2.1 ± 0.4
Lung	5.8 ± 1.6	2.3 ± 0.3	3.6 ± 1.0	2.3 ± 1.0	3.4 ± 0.7	3.8 ± 1.6
Muscle	—	0.4 ± 0.1	0.5 ± 0.1	0.3 ± 0.1	—	0.3 ± 0.1
Pancreas	5.5 ± 1.1	2.3 ± 0.2	3.4 ± 1.3	1.9 ± 0.8	5.2 ± 0.6	1.6 ± 0.3
Spleen	5.2 ± 1.1	1.7 ± 0.5	2.8 ± 1.1	1.8 ± 0.8	4.4 ± 2.1	1.1 ± 0.3
Thyroid	0.4 ± 0.2	—	—	—	0.2 ± 0.1	—-

*The intestines were measured without their contents.
